# Disruption of Circadian NRF2 Expression and Its Impact on Pain Sensitivity in Diabetes Peripheral Neuropathy

**DOI:** 10.1155/prm/5510019

**Published:** 2025-09-27

**Authors:** Wanshi Liang, Yali Song, Zebin Yang, Ronghao Luo, Xinran Li, Yihao Guo, Yang Zhang, Yan Liu, Le Li

**Affiliations:** ^1^Department of Anesthesiology, Zhujiang Hospital, Southern Medical University, Guangdong, Guangzhou 510282, China; ^2^Department of Anesthesiology, Hengqin Hospital, First Affiliated Hospital of Guangzhou Medical University (Guangdong-Macao In-Depth Cooperation Zone in Hengqin Central Hospitals), Guangdong, Zhuhai 519031, China; ^3^Division of Orthopaedic Surgery, Department of Orthopaedics, Southern Medical University, Nanfang Hospital, Guangdong, Guangzhou 510515, China

**Keywords:** chronotherapy, diabetic peripheral neuropathy, nuclear factor erythroid 2-related factor 2, reactive oxygen species

## Abstract

**Objective:** Diabetic peripheral neuropathy (DN) is a common complication of diabetes, characterized by symptoms that are milder during the day and worsen at night. This study aims to uncover the role of circadian NRF2 expression in dorsal root ganglia (DRG) in regulating pain sensitivity and explores its disruption on neuropathic pain.

**Method:** Male BKS.Cg-Lepr^db/db^/J (db/db) type 2 diabetic mice (T2DM) were used as a model for DN. Diurnal pain sensitivity in the mice was evaluated through force withdrawal threshold (FWT) and thermal withdrawal latency (TWL) at Zeitgeber Time (ZT) 2 and ZT14 from 8 to 16 weeks of age. Sciatic nerve conduction velocity (SNCV) and oxidative stress levels in DRG were evaluated. Oxidative stress levels and antioxidant activity were assessed using fluorescent probe staining. NRF2 expression was evaluated through molecular and histological methodologies. The circadian regulatory genes (*Clock, Bmal1*), inflammatory factors (IL-6 and IL-10), and NRF2 target gene HO-1 were all detected by qRT-PCR. To directly investigate NRF2's role, AAV-mediated intrathecal injection was used to knock down NRF2 in DRG, disrupting its circadian rhythmicity.

**Results:** A significant diurnal variation in neuropathic pain sensitivity was observed in db/db mice, with increased pain sensitivity at ZT2 compared to ZT14. Elevated ROS levels were detected in the DRG of db/db mice, especially at ZT2. In db/+ mice, NRF2 showed diurnal rhythms with higher expression at ZT2, a pattern disrupted in db/db mice, accompanied by elevated ROS levels and inflammation in the DRG. NRF2 knockdown yielded distinct effects: in db/db mice, it further elevated ROS levels at ZT14, impaired antioxidant capacity, and imbalance between pro-inflammatory and anti-inflammatory factors without significantly altering pain sensitivity, whereas in db/+ mice, it reduced pain thresholds and induced diurnal variations in pain sensitivity.

**Conclusion:** The circadian rhythmicity of NRF2 in nondiabetic (db/+) mice is essential for maintaining the balance between anti- and pro-ROS, as well as inflammation, thereby preventing pain exacerbation and diurnal variations. Its disruption increases oxidative stress and inflammation, associated with induced diurnal pain sensitivity. In diabetic (db/db) mice, the loss of NRF2 rhythmicity exacerbates oxidative stress but minimally affects pain sensitivity, indicating a ceiling effect in pain sensitivity. These findings highlight NRF2 rhythmicity as a potential chronotherapeutic target for managing diabetic neuropathy.

## 1. Introduction

Diabetic neuropathy, a prevalent complication of diabetes mellitus, affects approximately 50% of all diabetic patients globally and manifests as chronic pain, sensory loss, and motor impairment, severely impairing quality of life [1]. Notably, clinical observations highlight a circadian pattern in diabetic peripheral neuropathy (DN) symptom severity, with patients frequently reporting intensified pain during nocturnal periods [2]. Understanding the mechanisms behind this diurnal variation is crucial for improving therapeutic approaches.

While oxidative stress and inflammation are established drivers of DN pathogenesis [3], the mechanisms underlying temporal fluctuations in pain remain poorly characterized. Oxidative stress levels, regulated by endogenous circadian rhythms, exhibit diurnal oscillations in both physiological and pathological states, with higher levels typically observed at night [4]. This fluctuation may exacerbate neuropathic pain in DN patients during nighttime.

Nuclear factor erythroid 2-related factor 2 (NRF2), a crucial regulator of cellular antioxidant responses, coordinates the transcription of cytoprotective genes to counteract oxidative and inflammatory insults [5]. In diabetic, NRF2 dysfunction has been implicated in impaired redox balance, promoting neuronal injury and peripheral neuropathy [6]. However, whether NRF2 activity itself follows circadian rhythms in dorsal root ganglia (DRG)—critical hubs for pain signal transmission—and how such rhythmicity influences diurnal pain patterns in DN remains unexplored.

This study explores NRF2's involvement in the circadian variation of neuropathic pain in a mouse model of type 2 diabetes. By analyzing pain responses, oxidative stress, and NRF2 expression at different times of the day, we aim to uncover mechanisms driving DN's diurnal pain patterns. Our findings may offer new insights into DN management and highlight novel targets for chronotherapeutic interventions.

## 2. Material and Methods

### 2.1. Animals and Housing Conditions

Male db/db (BKS.Cg-Lepr^db/db^/J) mice and db/+ (BKS.Cg-Lepr^db/+^/J) mice as the control group were procured from Jakson's Lab. Mice were housed in under specific pathogen-free (SPF) conditions with controlled temperature(22 ± 2°C), humidity(40%–70%), and a 12/12 light-dark cycle (light phase: 07:00-19:00, ZT0-ZT12; dark phase: 19:00-07:00). Body weight and blood glucose levels were monitored weekly via tail vein sampling using a glucometer (FreeStyle Optium Neo H, Abbott, UK). This research protocol received approval from the National Animal Care and Use Committee at Zhujiang Hospital of Southern Medical University, Guangzhou, China (Approval No. LAEC-2022-034). All animal procedures adhered to the guidelines outlined in the National Research Council's Guide for the Care and Use of Laboratory Animals.

### 2.2. Intrathecal Injection of Virus in Mice

Eight-week-old male db/db and db/+ mice were anesthetized with isoflurane (4% induction and 2% maintenance) and placed in a stereotaxic frame for prone positioning following shaving and aseptic preparation of the lumbar region. Using a Hamilton syringe with a 30-gauge needle, 10 μL of 3.5 × 10^11^ genome copies (GC) of AAV9-CaMKIIα-shNRF2 (AAV9-shNRF2) was injected into the intrathecal space between the L4 and L5 vertebrae, confirmed by a tail flick response. The injection was performed over 2 min, and the needle was held in place for 30 s to prevent backflow. Control mice received AAV9-CaMKIIα-scramble (AAV9-sc). Animals were monitored daily postinjection and analyzed to allow for viral transduction and gene knockdown.

### 2.3. Blood Glucose and Body Weight Measurements

To assess circadian metabolic variations, we conducted measurements at two specific time points known as ZT2 (2 h after the start of the light phase) and ZT14 (2 h after the start of the dark phase). Tail vein blood was collected under aseptic conditions at these designated time points. Blood glucose levels were assessed using a glucometer (FreeStyle Optium Neo H, Abbott, UK), while body weights were carefully measured at ZT2 and ZT14 using a digital scale with a precision of 0.01 g.

### 2.4. Force Withdrawal Threshold (FWT)

Circadian variations in mechanical nociception were evaluated using Von Frey filaments (NC12775-99, Touch-Test, USA). Mice were placed on a wire mesh table (mesh size: 2 × 2 mm), and the lateral surface of the right hind paw was stimulated. Mechanical allodynia was assessed using the Dixon up-and-down method. Paw withdrawal prompted the use of a filament with lower force, while no response led to the application of a filament with higher force. The 50% paw withdrawal threshold (PWT, in grams) was calculated using a reported formula [7] and recorded as the 50% FWT.

### 2.5. Thermal Withdrawal Latency (TWL)

Mice were acclimated in a plexiglass chamber for 15 min prior to nociceptive stimulation using a hot plate (35,150, Ugo Basile, IT). TWL was defined as the time from the onset of heat stimulation (52°C) to paw withdrawal or avoidance behavior. To prevent sensitization, a 10-min interval was maintained between stimulations, and the same area of the paw was consistently stimulated in each trial. Latency measurements were averaged over three consecutive trials for each mouse.

### 2.6. Measurement of Sciatic Nerve Conduction Velocity (SNCV)

Mice were anesthetized with pentobarbital sodium (50 mg/kg) for surgery to expose the sciatic nerve. Nerve potentials were recorded using an electrostimulator and ring electrodes. Data analysis was performed using a biological signal acquisition system (MP160, ERS100C, BIOPAC, USA).

### 2.7. DRG Tissue Isolation

For the isolation of DRG tissue, mice were deeply anesthetized with an intraperitoneal injection of sodium pentobarbital (50 mg/kg) to ensure minimal discomfort and rapid anesthesia induction. Following perfusion with cold PBS, the mice were decapitated, and the vertebral column was swiftly excised. Under a dissecting microscope, the vertebral column was delicately opened to access the DRGs, and small rounded structures located bilaterally adjacent to the spinal cord in the lumbar (L4-L6) region. The DRGs were meticulously dissected using fine forceps and micro-dissecting scissors.

### 2.8. Reactive Oxygen Species (ROS) Determination

To assess ROS levels in mouse DRGs, tissues were embedded in optimal cutting temperature compound (OCT, OCT-4583, Sakura, Japan) and cryosectioned at 12 μm thickness. ROS detection was performed using a commercial frozen-section ROS detection kit (BB-470516, BestBio Science, Shanghai, China) following the manufacturer's protocol. Fluorescent signals were acquired using a fluorescence microscope (excitation: 535 nm; emission: 610 nm) with consistent acquisition parameters across all samples. Imaging and analysis of the sections were conducted using Image J software.

### 2.9. Quantitative Real-Time PCR

After washing the DRG tissues twice with ice-cold PBS, total mRNA was extracted using Trizol reagent (AG21102, Accurate Biotechnology, Hunan, China) following the manufacturer's instructions. RNA concentration was determination by measuring the absorbance at 260 nm. The total RNA was reverse transcribed into cDNA using high-capacity reverse transcriptase and 1 μM oligo (dT) (AG11706, Accurate Biotechnology, Hunan, China). Gene-specific primers for mice were used as shown in Table 1, and the cDNA was normalized to β-actin levels. SYBR Premix Ex Taq (AG11701, Accurate Biotechnology, Hunan, China) was utilized in the experiment and conducted inquantitative PCR system (LightCycler 480, Roche, USA). All samples were run in triplicate and analyzed using the 2^−ΔΔCt^ method, following established protocols [7].

### 2.10. Western Blot Analysis

To analyze NRF2 expression levels in DRG, tissue homogenates were lysed in RIPA buffer (P0013C, Beyotime, Shanghai, China), and protein concentrations were quantified using a BCA assay kit (P0010, Beyotime, Shanghai, China). Equal amount of protein (35 μg) was separated by SDS-PAGE at 120 V for 80 min and transferred onto PVDF membranes (Millipore, Billerica, MA, USA). The membranes were blocked in 5% nonfat milk for 1 h at room temperature, followed by overnight incubation at 4°C with primary antibodies (1:1000, NBP1-32822, Novus Biologicals, USA). After incubation, the membranes were probed with species-specific horseradish peroxidase-conjugated secondary antibodies (1:5000, G21234, Invitrogen, USA) for 1 h at room temperature. Protein bands were visualized using an ECL chemiluminescence substrate kit (WBKLS0500, Millipore, Billerica, MA, USA), and images were captured with a Bio-Rad imaging system (CFX Manage, Bio-Rad, USA). Protein levels in different experimental groups were presented as percentages relative to the control group.

### 2.11. Immunofluorescent (IF) Analysis

DRG tissue samples were fixed by immersion in 4% paraformaldehyde in phosphate buffer, followed by cryoprotection in 30% sucrose-PBS solution for 24 h. Cryosections of 12 μm thickness were prepared with a cryostat and mounted onto gelatin-coated slides. Blocking was performed using 1% bovine serum albumin containing 0.3% Triton X-100 for 1 h. The sections were then incubated overnight at 4°C with primary antibodies: rabbit anti-NRF2 (1:200, NBP1-32822, Novus Biologicals, USA) and rabbit anti-NeuN (1:200, ab177487, Abcam, UK). After primary antibody incubation, the sections were treated with Alexa Fluor® 488-conjugated and Alexa Fluor® 594-conjugated goat anti-rabbit IgG H&L secondary antibodies (ab96899-500ug, ab150080-500ug, Abcam, UK) for 1 h at room temperature. Nuclei were counterstained with DAPI (ab104139, Thermo Fisher, USA). Finally, all sections were examined, and images were captured using a fluorescence microscope.

### 2.12. Measurement of Antioxidant Capacity

DRG tissues were homogenized in precooled lysis buffer and centrifuged at 12,000 rpm for 10 min at 4°C to collect the supernatant. The activities of total superoxide dismutase (T-SOD), total antioxidant capacity (T-AOC), glutathione peroxidase (GSH-PX) activity and catalase (CAT) activity were measured using assay kits (A001-3-2, A015-2-1, A005-1-2, and A007-2-1 Nanjing Jiancheng Bioengineering Institute, China), following the manufacturer's instructions. T-SOD activity was determined using the water-soluble tetrazolium salt (WST-1) method, which measures the reduction of WST-1 by superoxide anions at 450 nm. T-AOC levels were quantified using the 2,2′-azino-bis(3-ethylbenzothiazoline-6-sulfonic acid) (ABTS) method, with absorbance measured at 520 nm. CAT activity was measured by monitoring the rate of H_2_O_2_ decomposition, reflected by the decrease in absorbance at 240 nm due to H_2_O_2_ consumption. GSH-GPx activity was measured by monitoring GSH depletion in absorbance at 240 nm. All enzyme activities were calculated based on the manufacturer's protocols and expressed as U/mg or U/pg protein.

## 3. Results

### 3.1. The Diurnal Difference in Blood Glucose

**Figure 1:**
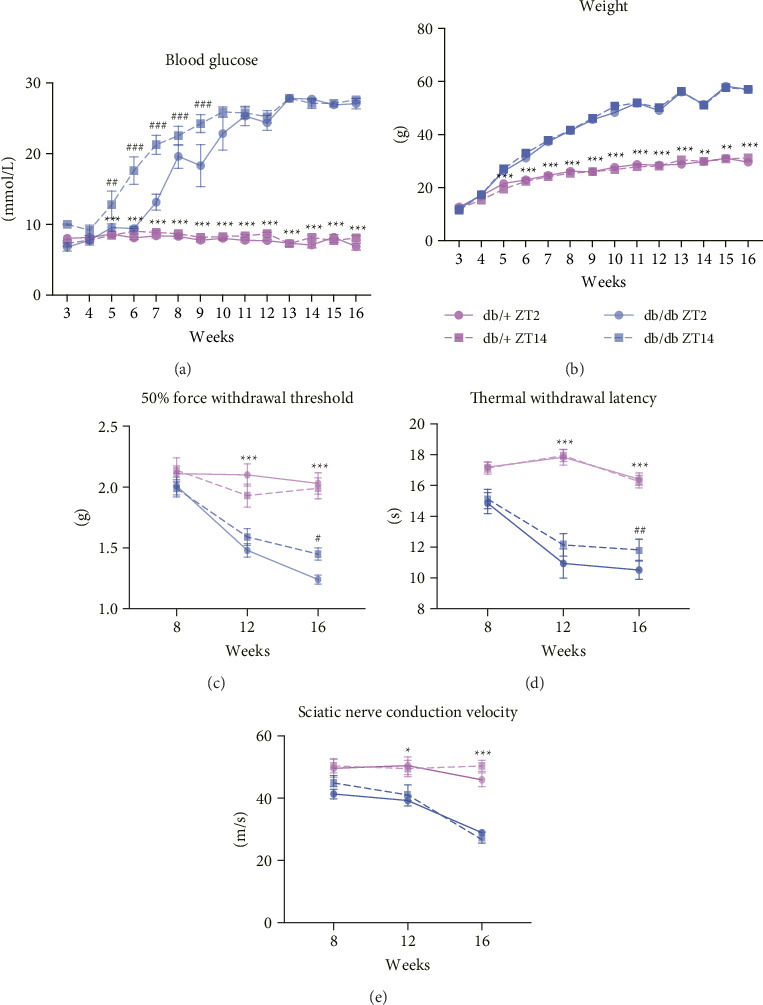
Blood glucose and weight (a) Comparison of blood glucose levels between db/db mice and db/+ mice at ZT2 and ZT14 time points at different ages; (b) comparison of body weight between db/db mice and db/+ mice at ZT2 and ZT14 at different ages; (c) 50% paw withdrawal threshold; (d) thermal paw withdrawal latency; and (e) sciatic nerve conduction velocity at ZT2 and ZT14 in male db/+ and db/db mice at 8, 12, and 16 weeks of age. All data are expressed as mean ± SE, *n* = 10, ^∗∗^*p* < 0.01, ^∗∗∗^*p* < 0.001, ^∗^the statistical significance of db/+ group versus db/db group; ^#^*p* < 0.05. ^##^*p* < 0.01, ^###^*p* < 0.001, # indicates the statistical significance of ZT2 time point versus ZT14 time point in db/db group.

Blood glucose levels in db/db mice became significantly higher than those in db/+ mice from the fifth week. At the same time, db/db mice showed diurnal variations, with levels at ZT14 significantly higher than at ZT2 (Figure 1(a)). After the 10th week, some db/db mice exceeded the glucometer's measurement limit (27.8 mmol/L), indicating the need for improved measurement methods.

### 3.2. Diurnal Variation in DN Symptoms

To assess diurnal variations in DN symptoms, the 50% FWT, thermal pain threshold (TWL), and SNCV in db/db and db/+ mice at ZT2 and ZT14 aged 8, 12, and 16 weeks were measured.

As illustrated in Figure 1(c), significant differences in the 50% FWT were observed between db/+ and db/db mice at 12 and 16 weeks of age. Specifically, db/db mice exhibited a markedly lower 50% FWT compared to db/+ mice, indicating heightened mechanical allodynia in the diabetic mice. Furthermore, at 16 weeks, db/db mice demonstrated a significant diurnal variation in the 50% FWT between ZT2 and ZT14. The 50% FWT at ZT2 was significantly lower than that at ZT14.

Similarly, as shown in Figure 1(d), db/db mice demonstrated a significantly shorter TWL than db/+ mice starting from 12 weeks of age, reflecting heightened thermal pain sensitivity. At 16 weeks, TWL at ZT2 in db/db mice was lower than that at ZT14, suggesting increased pain sensitivity during the early light phase (inactive phase in rodents).

In contrast, as shown in Figure 1(e), SNCV in db/db mice was lower than that in db/+ mice at 12 weeks of age. However, no significant diurnal variation in SNCV was observed at any age in db/db mice.

### 3.3. Diabetic Conditions Result in Diurnal Variation of ROS Level

**Figure 2:**
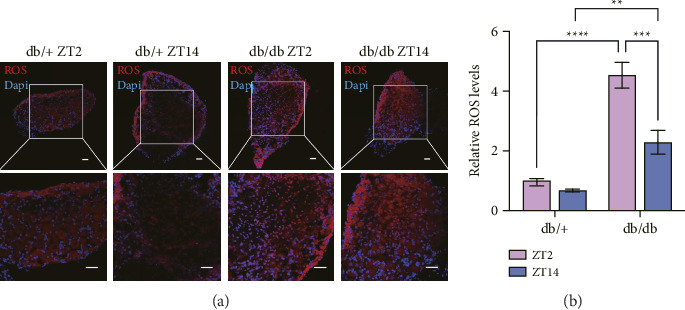
Diabetic conditions result in diurnal variation of ROS level. (a) Representative of ROS staining results after cryostat sectioning of DRG, observed under a 10× objective; (b) quantitative analysis ROS after normalization. All data are expressed as mean ± SD, *n* = 5–7, ^∗∗^*p* < 0.01, ^∗∗∗^*p* < 0.001, ^∗∗∗∗^*p* < 0.0001, ^∗^the statistical significance of different groups.

At 16 weeks of age, there was no significant diurnal variation in oxidative stress levels in db/+ mice, whereas db/db mice exhibited significantly higher oxidative stress levels at ZT2 compared to both db/+ mice and db/db mice at ZT14 (Figure 2).

### 3.4. Diurnal Variation of NRF2 in Diabetes Condition

**Figure 3:**
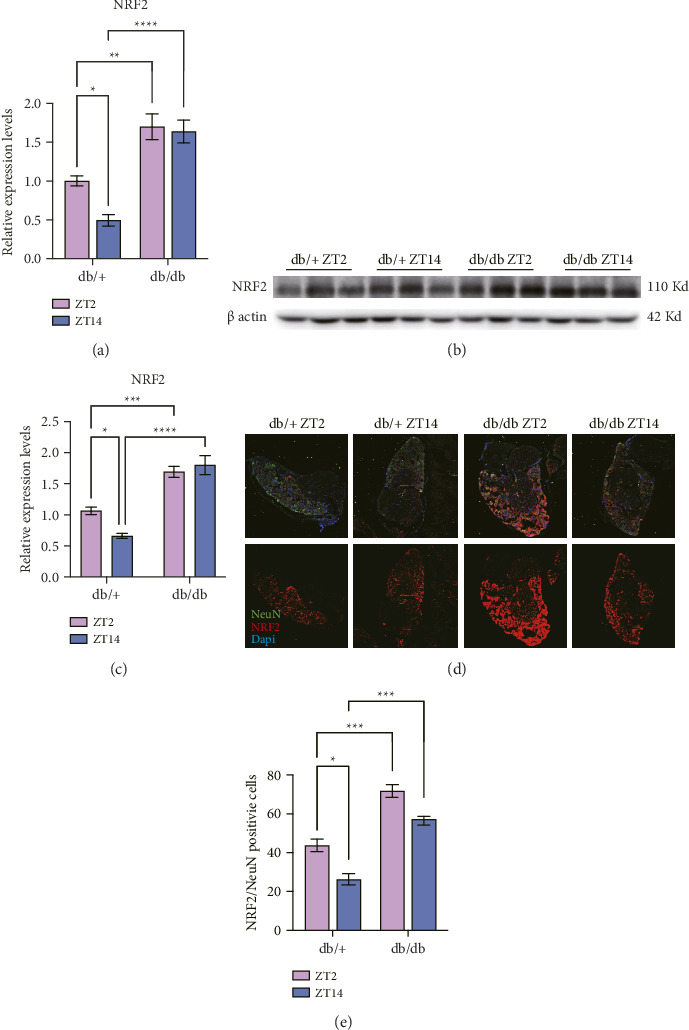
Diurnal variation of NRF2 in diabetes condition. (a) NRF2 mRNA expression levels of db/db and db/+ DRG at 16 weeks of ZT2 and ZT14; (b & c) NRF2 protein of db/db and db/+ DRG at 16 weeks of ZT2 and ZT14; (d) representative of NRF2 staining results after cryostat sectioning of DRG, observed under a 10× objective; (e) quantitative analysis NRF2 after normalization. All data are expressed as mean ± SE, *n* = 10, ^∗^*p* < 0.05, ^∗∗^*p* < 0.01, ^∗∗∗^*p* < 0.001, ^∗∗∗∗^*p* < 0.0001. ^∗^The statistical significance of different group.

At 16 weeks, NRF2 mRNA (Figure 3(a)) and protein (Figure 3(b)) levels in DRG tissues were significantly higher in db/db mice than in db/+ mice at both ZT2 and ZT14. In db/+ mice, NRF2 expression showed diurnal rhythms with higher levels at ZT2, while this rhythm was absent in db/db mice. Immunofluorescence revealed that NRF2 colocalized with the neuronal marker NeuN and displayed a punctate nuclear distribution in db/+ mice, whereas it appeared diffusely expressed in db/db mice. Additionally, NRF2 fluorescence intensity in db/+ mice was higher at ZT2, but this rhythmicity was lost in db/db mice, despite their overall higher NRF2 levels (Figures 3(d), 3(e)).

### 3.5. NRF2 Impacts on Diurnal Variation of DN Symptoms

**Figure 4:**
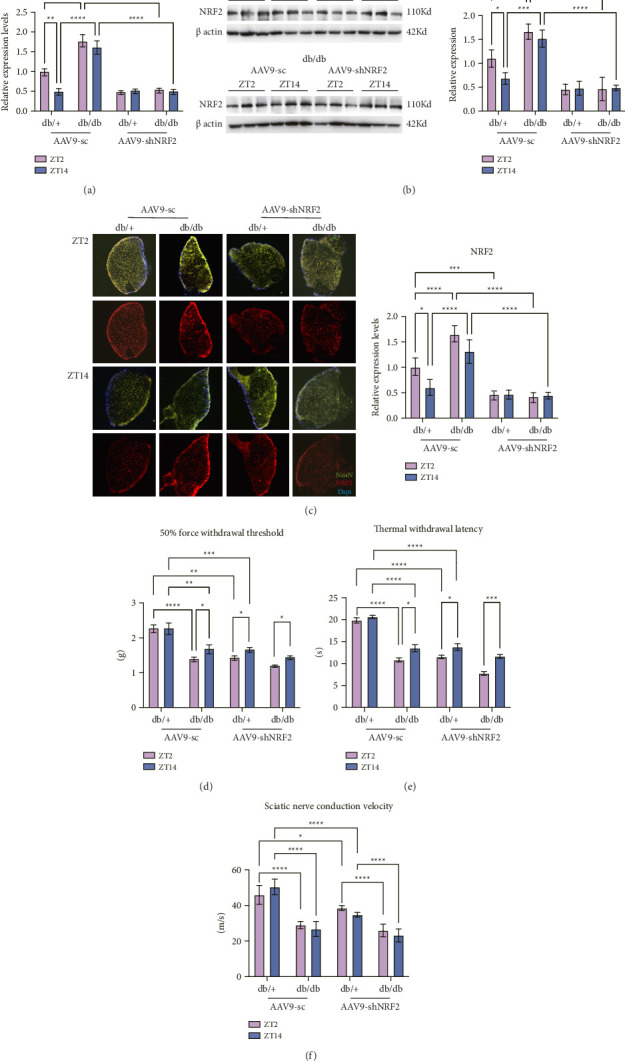
NRF2 impacts on diurnal variation of diabetic neuropathy symptoms. (a) NRF2 mRNA expression levels of db/db and db/+ DRG; (b) NRF2 protein levels of db/db and db/+ DRG. Representative Western blot for NRF2 (left) and corresponding quantitative analysis (right); (c) immunofluorescence assays of NRF2 protein using fluorescence microscope (observed under a 10× objective). Representative images for NRF2 (left) and corresponding quantitative analysis (right); (d) 50% paw withdrawal threshold; (e) thermal paw withdrawal latency; and (f) sciatic nerve conduction velocity in male db/+ and db/db mice after NRF2 knockdown. All data are expressed as mean ± SE, *n* = 3–6, ^∗^*p* < 0.05, ^∗∗^*p* < 0.01, ^∗∗∗^*p* < 0.001, ^∗∗∗∗^*p* < 0.0001, ^∗^the statistical significance of different groups. AAV9-shNRF2 represents mice with NRF2 knockdown, while AAV9-sc represents control mice.

Diurnal rhythmicity of NRF2 expression was first examined at the mRNA level (qPCR, Figure 4(a)). In db/+ mice treated with AAV9-sc, NRF2 mRNA levels peaked at ZT2, demonstrating a robust diurnal rhythm (*p* < 0.001). By contrast, db/db mice showed a blunted NRF2 mRNA rhythm, with no significant difference between ZT2 and ZT14. Genetic knockdown of NRF2 via AAV9-shNRF2 abolished the diurnal rhythm in both db/+ and db/db groups, as NRF2 mRNA levels remained low and comparable between ZT2 and ZT14 in AAV9-shNRF2-treated mice (*p* > 0.05 for ZT2 vs. ZT14 in shNRF2 groups). Consistent with mRNA data, NRF2 protein levels (Western blot, Figure 4(b)) followed a similar diurnal pattern: db/+ mice displayed higher NRF2 protein at ZT2 than ZT14 (*p* < 0.01), whereas db/db mice showed no ZT-dependent variation. AAV9-shNRF2 treatment potently reduced NRF2 protein abundance in both genotypes and time points, with no residual diurnal oscillation (*p* > 0.05 for ZT2 vs. ZT14 in shNRF2 groups). These results demonstrate that NRF2 expression is diurnally regulated in db/+ DRG neurons, and this regulation is lost in diabetic db/db mice and further disrupted by NRF2 knockdown.

Immunofluorescence (Figure 4(c)) confirmed NRF2 neuronal localization (colocalization with NeuN) and diurnal rhythm in db/+ mice (*p* < 0.001). db/db mice displayed lower NRF2 levels without rhythmicity, and shNRF2 treatment abolished the rhythm (*p* > 0.05).

In db/+ mice treated with AAV9-sc, db/db mice exhibited significantly reduced 50% paw withdrawal threshold (FWT) (Figure 4(d)) and thermal paw withdrawal latency (TWL) (Figure 4(e)), indicating pronounced mechanical allodynia and thermal hyperalgesia, respectively. AAV9-shNRF2 treatment further decreased FWT and TWL in db/+ mice (*p* < 0.05), confirming that NRF2 deficiency exacerbates pain sensitivity under physiological conditions.

Strikingly, db/db mice receiving AAV9-shNRF2 treatment displayed robust diurnal oscillations in both FWT and TWL, with significantly lower thresholds at ZT2 compared to ZT14 (*p* < 0.01). Quantitative analysis revealed that the ZT2-ZT14 difference in nociceptive thresholds was amplified by 1.8-fold in shNRF2-treated db/db mice relative to AAV9-sc controls (*p* < 0.001), demonstrating that NRF2 knockdown potentiates diabetes-induced circadian disruption of pain sensitivity.

Consistent with behavioral findings, SNCV was markedly reduced in db/db mice compared to db/+ mice under AAV9-sc treatment (*p* < 0.001, Figure 4(f)), confirming DN-induced functional impairment. While AAV9-shNRF2 treatment further decreased SNCV in db/+ mice (*p* < 0.05), no additional reduction was observed in db/db mice following NRF2 knockdown (*p* > 0.05 vs. AAV9-sc group). This selective vulnerability suggests that diabetes-associated nerve damage may saturate the functional deficits, masking the additive effects of NRF2 deficiency.

### 3.6. NRF2-Related Circadian Regulatory

**Figure 5:**
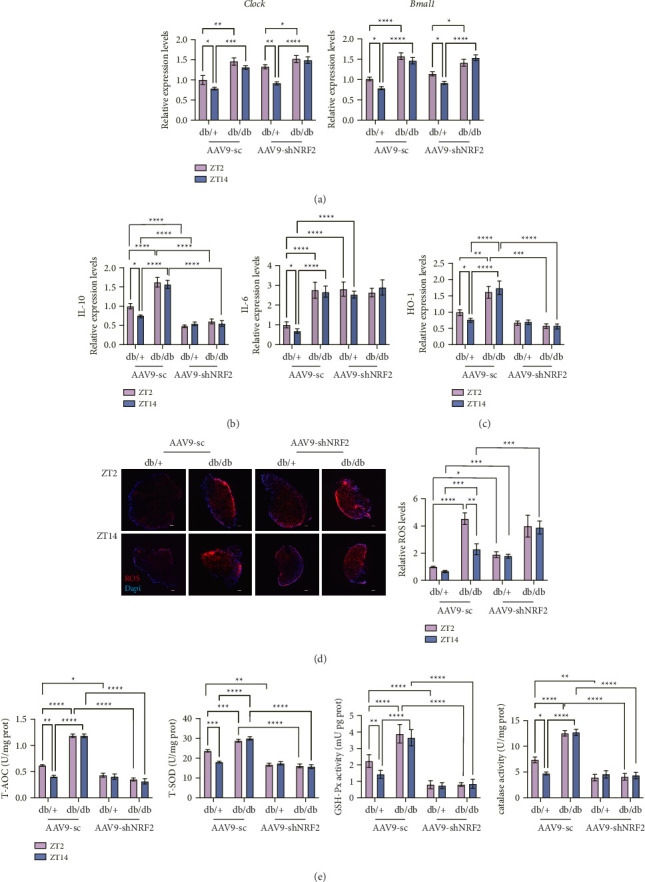
NRF2 in diurnal variation of antioxidant capacity. (a) mRNA expression of *Clock* and *Bmal1* in db/+ and db/db DRG transduced with AAV9-sc or AAV9-shNRF2 at ZT2/ZT14. (b-c) IL-10 (left), IL-6 (middle), and HO-1(right) mRNA level. (d) ROS levels in DRG (representative images, left) and quantification (right). Scale bar = 100 μm. (e) The activities of total antioxidant capacity (T-AOC), total superoxide dismutase (T-SOD), glutathione peroxidase (GSH-PX), and catalase (CAT). All data are expressed as mean ± SE, *n* = 6, ^∗^*p* < 0.05, ^∗∗^*p* < 0.01, ^∗∗∗^*p* < 0.001, ^∗∗∗∗^*p* < 0.0001, ^∗^the statistical significance of different groups. AAV9-shNRF2 represents mice with NRF2 knockdown, while AAV9-sc represents control mice.

The expression of *Clock* and *Bmal1* mRNA exhibited a clear circadian rhythm in db/+ mice, with higher levels observed at ZT2 compared to ZT14 (Figure 5(a)). This rhythmic pattern was consistent with the circadian rhythm of *Nrf2*. However, in db/db mice, the circadian rhythm of *Clock* and *Bmal1* was completely abolished, and their expression levels were significantly elevated. Notably, in *Nrf2* knockdown (AAV9-shNRF2) db/db and db/+ mice, the expression patterns of *Clock* and *Bmal1* were unchanged compared to *Nrf2*-intact mice. These findings suggest that *Nrf2* does not directly regulate the expression of *Clock* and *Bmal1*. Instead, these genes likely represent more fundamental, upstream regulators of circadian rhythms, whose expression is independent of NRF2 activity.

### 3.7. NRF2 Regulates Diurnal Oscillations in Pro- and Anti-Inflammatory Markers

In db/+ mice, pro-inflammatory (IL-6) and anti-inflammatory (IL-10) cytokines exhibited synchronized diurnal rhythms, peaking synchronously at ZT2 (Figure 5(b)). This rhythmic balance suggests a tightly regulated inflammatory response aligned with the circadian clock. In contrast, db/db mice displayed elevated basal levels of both IL-6 and IL-10, but their diurnal oscillations were completely abolished, indicating a disrupted inflammatory homeostasis. Notably, NRF2 knockdown (AAV9-shNRF2) further exacerbated these disruptions: IL-6 Levels increased significantly both in db/+ (vs. db/+ AAV9-sc) and db/db (vs. db/db AAV9-sc) mice across all time points, with complete loss of rhythmicity. IL-10 levels decreased markedly both in db/+ (vs. db/+ AAV9-sc) and db/db (vs. db/db AAV9-sc) mice, and the ZT2 peak was abolished. These findings demonstrate that NRF2 is essential for maintaining the diurnal balance between pro- and anti-inflammatory responses, and its deficiency disrupts this equilibrium in diabetic conditions.

### 3.8. HO-1 as a Downstream Effector of NRF2

In db/+ mice, *Ho-1* mRNA expression followed a clear diurnal rhythm, peaking at ZT2 (Figure 5(c)), which paralleled the rhythmicity of NRF2 (Figure 5(a)). In db/db mice, *Ho-1* expression was constitutively elevated but lost its rhythmicity (*p* > 0.05 for ZT2 vs. ZT14), suggesting a dysregulated compensatory response to chronic inflammation. NRF2 knockdown (AAV9-shNRF2) further suppressed *Ho-1* expression both in db/+ (vs. db/+ AAV9-sc) and db/db (vs. db/db AAV9-sc) mice across all time points and abolished diurnal oscillation (*p* > 0.05 for ZT2 vs. ZT14). This direct correlation between NRF2 activity and *Ho-1* rhythmicity confirms HO-1 as a functional downstream target of NRF2 in the inflammatory clock mechanism.

### 3.9. NRF2 in Diunal Variation of Antioxidant Capacity

In db/+ mice, ROS levels remained consistently low without significant diurnal fluctuation, while antioxidant capacity (T-AOC, T-SOD, GSH-Px, and CAT) peaked at ZT2, suggesting a time-dependent adaptation for efficient ROS clearance (Figures 5(d), 5(e)). This rhythmic antioxidant pattern indicates a synchronized defense mechanism against diurnal oxidative challenges in physiological conditions. In db/db mice, ROS levels were significantly elevated at both ZT2 and ZT14 (*p* < 0.001 vs. db/+) and exhibited a disrupted rhythmicity, with a paradoxical peak at ZT2 (vs. ZT14, *p* < 0.01). Concurrently, the diurnal oscillations of all antioxidant enzymes (T-AOC, T-SOD, GSH-Px, and CAT) were abolished, reflecting a compromised redox defense system in diabetes.

NRF2 deficiency (AAV9-shNRF2) disrupted redox homeostasis in both genotypes by abolishing diurnal antioxidant rhythms and exacerbating oxidative stress. In db/+ mice, NRF2 knockdown abolished the diurnal rhythms of T-AOC, T-SOD, GSH-Px, and CAT (all *p* > 0.05 for ZT2 vs. ZT14), with significant activity reductions at ZT2 compared to AAV9-sc controls (*p* < 0.05 for all enzymes). Concurrently, ROS levels increased at ZT2 (*p* < 0.01), indicating impaired ROS clearance. In db/db mice, pre-existing disruptions in antioxidant rhythms (lost diurnal oscillations for all enzymes) were further exacerbated by NRF2 knockdown, with enzyme activities declining compared to db/db AAV9-sc control levels at both ZT2 and ZT14 (*p* < 0.0001 for all enzymes). ROS levels in db/db mice were further elevated compared to db/db AAV9-sc controls at ZT14 (*p* < 0.001), reflecting severe redox imbalance.

## 4. Discussion

The study revealed several key findings: (i) The diurnal variation in neuropathic pain sensitivity in db/db mice is timing-dependent, with increased pain sensitivity observed at ZT2 compared to ZT14. This variation is associated with elevated ROS levels at ZT2, suggesting a link between oxidative stress and pain sensitivity. (ii) NRF2 dysfunction contributes to oxidative stress dysregulation and diurnal pain variation in db/db mice. NRF2 rhythmicity was disrupted in db/db mice, leading to impaired antioxidant capacity and exacerbated ROS levels, while NRF2 knockdown further increased oxidative stress but had limited impact on pain sensitivity, likely due to a ceiling effect. (iii) NRF2 rhythmicity prevents diurnal pain variation and maintains oxidative balance in db/+ mice. In db/+ mice, NRF2 expression showed intact circadian rhythmicity, with higher expression at ZT2, which reduced ROS levels and prevented diurnal pain variation. However, NRF2 knockdown disrupted this balance, inducing diurnal pain sensitivity with heightened pain at ZT2.

Diurnal variations in pain hypersensitivity are common in chronic pain disorders [8] and have also been confirmed in patients with cancer [9], rheumatoid arthritis [10], fibromyalgia [11], and multiple sclerosis [12]. A previous human study also suggested that the pain intensity of DN exacerbates at night [13]. The results of our study corroborate the clinical observations that DN pain exhibits diurnal variation [2], with higher pain sensitivity during the light phase in db/db mice. These findings align with two previous reports observing the highest pain sensitivity during the light phase [14, 15]. In contrast, two studies reported the highest pain sensitivity exclusively during the dark phase [16, 17]. These varied results indicate that the timing of peak pain sensitivity in animal models can span both light and dark phases. This variability underscores the complexity of circadian influences on neuropathic pain and highlights the need for further research to understand the underlying mechanisms.

The imbalance between oxidative stress and antioxidant capacity are well-documented factors in the pathogenesis of DN [18]. When considering ROS and antioxidant capacity together, it is evident that db/db mice exhibit significantly elevated ROS levels, particularly at ZT2, indicating a diurnal variation. Although the antioxidant capacity in db/db mice is also higher compared to db/+ mice, the rhythmic pattern observed in db/+ mice disappears, suggesting antioxidant capacity that does not align with the ROS levels. This observation is consistent with the notion that circadian rhythms regulate not only oxidative stress levels but also the expression and activity of antioxidant enzymes, which play a critical role in maintaining redox homeostasis [19]. In the DRG of db/+ mice, the diurnal variation in ROS levels is accompanied by corresponding changes in antioxidant capacity, maintaining a balance that prevents oxidative damage. In contrast, although the antioxidant capacity in the DRG of db/db mice is elevated, it fails to adapt to the heightened ROS levels, leading to oxidative–antioxidative imbalance and subsequent neuronal damage, which may underlie the observed neuropathic pain patterns. Furthermore, the diurnal variation in the oxidative–antioxidative balance is closely linked is closely related to the activity rhythms of nocturnal animals like mice, which are more active during the night [20]. Increased activity during the dark phase is associated with higher metabolic rates, potentially leading to fluctuations in ROS production and antioxidant activity [21]. These findings highlight the importance of considering the timing of therapeutic interventions to restore the oxidative–antioxidative balance in the context of DN, as circadian rhythms may significantly influence treatment efficacy.

NRF2, a critical transcription factor involved in the antioxidant response [22], was found to exhibit distinct roles in db/+ and db/db mice. In db/+ mice, NRF2 displayed clear diurnal rhythmicity, with higher expression levels at ZT2, which coordinated antioxidant defenses by regulating its target genes involved in detoxification [23] and oxidative stress response [24]. This rhythmic NRF2 activity contributed to maintaining the oxidative–antioxidative balance, preventing oxidative damage, and avoiding diurnal variations in pain sensitivity [25].

In contrast, the db/db mice exhibited a loss of NRF2 rhythmicity, with consistently elevated NRF2 levels that lacked significant diurnal variation. Although NRF2 expression was increased, its disrupted rhythmicity limited its effectiveness in coordinating antioxidant defenses. This dysfunction contributed to the imbalance between oxidative stress and antioxidant capacity, leading to heightened ROS levels and increased pain sensitivity, particularly during the early light phase. Interestingly, NRF2 knockdown in db/db mice resulted in further increases in ROS levels at ZT14 but did not exacerbate pain sensitivity. This finding suggests that the relationship between oxidative stress and neuropathic pain may not be linear and that other compensatory mechanisms, independent of NRF2, might be involved in modulating pain perception. For instance, it is possible that the pain response has already reached a “ceiling effect” in db/db mice, where additional oxidative stress no longer translates into increased pain.

Alternative mechanisms contributing to diurnal pain variation should also be considered. Inflammation is a well-documented factor in neuropathic pain [25]. In db/+ mice, pro-inflammatory (IL-6) and anti-inflammatory (IL-10) markers exhibited circadian peaks at ZT2, but this rhythmicity was lost in db/db mice, accompanied by elevated levels. NRF2 knockdown further disrupted this balance, abolishing rhythmicity while increasing IL-6 and reducing IL-10 levels, which, to some extent, reflects the association between low NRF2 levels and decreased anti-inflammatory capacity, as well as increased pro-inflammatory activity, similar to findings observed in related studies [26].

NRF2 also plays a pivotal role in regulating microglial activation during DN. Microglia, the primary immune cells in the nervous system, are highly sensitive to oxidative stress and inflammation [27]. Under diabetic conditions, increased oxidative stress drives microglia toward a pro-inflammatory (M1) phenotype, releasing cytokines like IL-1β and TNF-α, which worsen neuroinflammation and neuronal damage [28]. NRF2 mitigates this by enhancing antioxidant defenses through genes such as HO-1, limiting excessive microglial activation [29].

Circadian regulators such as *Clock*, *Bmal1* and *Per3* have been implicated in modulating oxidative stress and inflammatory pathways, which are critical in the progression of DN and other circadian-related disorders [30, 31]. Our findings reveal a hierarchical regulatory relationship among these genes and Nrf2, with important therapeutic implications: *Nrf2* does not directly regulate the expression of core circadian genes such as *Clock* and *Bmal1*. *Clock* and *Bmal1*, as primary circadian regulators, exhibit rhythmic expression in normal conditions, high at ZT2 [32], which aligns with the circadian activity of *Nrf2*. *Clock* and *Bmal1* have been shown to regulate *Nrf2* expression through E-box-mediated transcriptional activation [22]. However, in the diabetic state, the rhythmicity of *Clock* and *Bmal1* is disrupted, and *Nrf2* knockdown does not affect their expression. This suggests that *Nrf2* acts as a downstream effector rather than a direct regulator of these core circadian genes. *Per3* is another core circadian component, although human studies showed no direct association with DN [31]. Instead, *Per3* polymorphisms show correlations with diabetes susceptibility [31] and infertility through circadian disruption [30]. These findings suggest that *Per3* may contribute to diabetic complications through mechanisms beyond direct *Nrf2* regulation, highlighting the need for further experimental studies to clarify the potential crosstalk between *Per3* and *Nrf2* in the pathogenesis of DN.

From a therapeutic perspective, targeting upstream circadian regulators like *Clock* and *Bmal1* or *Per3* could lead to unpredictable outcomes due to their widespread influence on the circadian network. This is supported by clinical data showing that *Per3* variants lack consistent association with DN [31], suggesting the complexity of circadian interventions.

In contrast*, Nrf2*, being a downstream effector primarily involved in antioxidant and anti-inflammatory responses, offers a more targeted and potentially safer intervention strategy. This approach could effectively modulate oxidative stress and inflammation without disrupting the broader circadian rhythms, providing a promising avenue for treating DN.

The findings of this study and related genetic evidence have important implications for the management of DN. The diurnal variation in pain sensitivity and oxidative stress suggests that timing of the administration of therapeutic agents to coincide with the body's circadian rhythms could enhance treatment efficacy. Chronotherapy, which involves synchronizing drug delivery with the body's biological clock, may offer a novel approach to managing DN pain more effectively. Targeting NRF2 pathways at specific times of the day could potentially restore redox balance and alleviate neuropathic pain.

Despite the promising findings, this study has several limitations. First, the use of mice as a model introduces challenges due to their nocturnal behavior, which differs from the diurnal nature of humans. Further, the absence of human subject data prevents direct translation to clinical contexts, particularly regarding circadian phase-specific interventions. However, the high conservation of core clock genes (e.g., *Clock*, *Bmal1*) [33] and pathways like NRF2 [34], along with extensive genetic tools, make mice a practical model for mechanistic studies. Clinical studies assessing diurnal NRF2 expression and pain sensitivity variations in diabetic patients, coupled with chronotherapeutic trials of time-dependent NRF2-targeted interventions, need to be conducted in the future. Second, the exact molecular mechanisms by which NRF2 influences diurnal pain variation require further elucidation. Future research should focus on exploring the interactions between NRF2 and other circadian regulators in DN and validating these findings in clinical settings. Lastly, the study lacks clinical human data, which limits its immediate translational relevance. We plan to extend our research to include clinical observations in human subjects in future studies, aiming to validate and enhance the applicability of our findings to human DN.

## 5. Conclusion

In conclusion, our study highlights the significant role of NRF2 in modulating the diurnal variation of neuropathic pain under diabetic conditions. The loss of NRF2 rhythmicity and the resulting imbalance in oxidative stress responses are key factors contributing to the increased pain sensitivity observed during the early light phase. Additionally, disrupted circadian regulation of inflammatory mediators, such as IL-6 and IL-10, further exacerbates this variation. These findings provide a foundation for exploring chronotherapeutic approaches that align treatment with circadian rhythms, offering a potential strategy for more effective management of diabetic neuropathic pain through targeted regulation of NRF2 pathways.

## Figures and Tables

**Table 1:** Primers required for qPCR experiments.

Primer name	Forward sequence (5′ to 3′)	Reverse sequence (5′ to 3′)
NRF2	TTCTTTCAGCAGCATCCTCTCCAC	ACAGCCTTCAATAGTCCCGTCCAG
β-actin	CTCTCTGTGGATTACGGAAAGAAG	GGTGGTGAGGATGGAATTGTAG
Clock	TGCTGGAAAGTGACTCCTTAACCC	TCATAGGTGGATGGCTCCTTTGGG
Bmal1	TGCCACCAATCCATACAC	TTCCCTCGGTCACATCCTAC
IL-10	TGGACTCCAGGACCTAGACAG	AGGACACCATAGCAAAGGGC
IL-6	AGTTGTGCAATGGCAATTCTGA	GGACTCTGGCTTTGTCTTTCTTGT
HO-1	CAGAAGAGGCTAAGACCGCC	CTCTGACGAAGTGACGCCAT

## Data Availability

The data that support the findings of this study are available from the corresponding author upon reasonable request.
